# The glucose-6-phosphatase system in cancer: from endoplasmic reticulum glucose-6-phosphate flux to stemness, immune escape, and therapeutic vulnerability

**DOI:** 10.3389/fonc.2026.1912198

**Published:** 2026-07-17

**Authors:** Bogdan Alexandru Danalache, Abdallah Fallah, Frédéric Mercier, Borhane Annabi

**Affiliations:** Laboratoire d’Oncologie Moléculaire, Département de Chimie, Université du Québec à Montréal, Montreal, QC, Canada

**Keywords:** cancer stemness, endoplasmic reticulum, G6PC, G6PT/SLC37A4, glucose-6-phosphatase, glucose-6-phosphate flux, immune escape, therapeutic vulnerability

## Abstract

The endoplasmic reticulum glucose-6-phosphatase (G6Pase) system, traditionally linked to hepatic and renal glucose homeostasis, is increasingly recognized as a regulator of intracellular glucose-6-phosphate (G6P) partitioning with broad relevance to cancer biology. Emerging evidence implicates its catalytic subunits (G6PC1-3) and associated transporters, particularly SLC37A4/G6PT, in redox control, calcium homeostasis, protein quality control, glycogen metabolism, autophagy, epithelial-mesenchymal transition, stemness, immune evasion, and therapy resistance. In several non-gluconeogenic cancers, elevated G6Pase-system activity is associated with aggressive phenotypes, whereas in liver and kidney, G6PC loss promotes metabolic disruption and dedifferentiation. This Review highlights how ER-directed G6P flux, rather than glucose production itself, may shape tumour behaviour and reveal context-specific therapeutic vulnerabilities. Importantly, the clinical targeting of this system remains at an early, largely preclinical stage, and the therapeutic opportunities discussed here should be regarded as hypotheses to be tested rather than established interventions.

## 1 Introduction

### 1.1 Why revisit the glucose-6-phosphatase system in oncology

A century after its formulation, the Warburg effect is still an important concept in the teaching of tumour metabolism: cancer cells preferentially convert glucose to lactate even in the presence of adequate oxygen, thereby supporting rapid biosynthesis and a permissive microenvironment (1). This cytosolic, glycolysis-centred view captures an essential feature of pathogenic and malignant metabolism, but it no longer suffices to explain the metabolic plasticity that allows tumours to survive hypoxia, nutrient limitation, proteotoxic stress and therapeutic pressure (2). Increasingly, attention has shifted from how much glucose a cancer cell consumes to how its central intracellular metabolite, glucose-6-phosphate (G6P), is partitioned among competing fates.

G6P sits at a true metabolic crossroads. It can be committed to glycolysis, diverted into the oxidative and non-oxidative pentose phosphate pathway (PPP) to generate NADPH and ribose (3), stored as glycogen (4), or transported into the endoplasmic reticulum (ER), where a dedicated phosphohydrolase system can release free glucose back into systemic blood circulation or into intracellular local pools (5). The enzymatic apparatus governing the last of these routes, the glucose-6-phosphatase (G6Pase) system, has historically been studied almost exclusively in the context of hepatic and renal gluconeogenesis and of type 1 glycogen storage diseases (GSD) (6). This article proposes that this same system, far from being a metabolic curiosity confined to gluconeogenic organs and neutrophil dysfunction leading to hyperlipidaemia, hyperuricaemia, lactic acidaemia, and growth retardation, operates in many tumours as a compartmentalized control point for G6P flux, and that its components are now emerging as both biomarkers and candidate therapeutic targets across a range of cancers.

**Figure 1:**
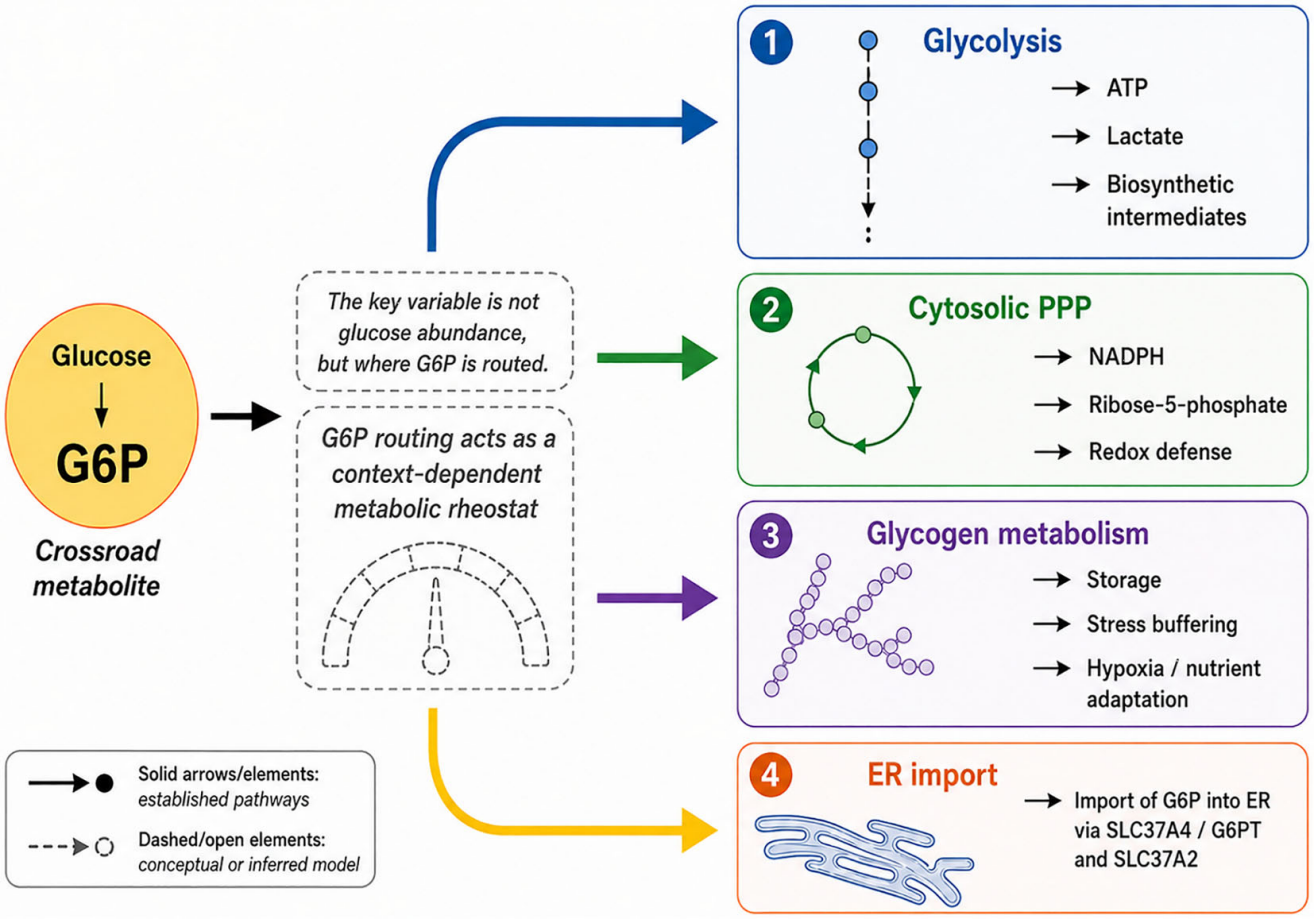
Competitive fates of glucose-6-phosphate and the ER-localized G6Pase flux module in cancer. Glucose-6-phosphate (G6P) occupies a central metabolic branch point in cancer cells. After glucose phosphorylation by hexokinases, cytosolic G6P can be routed toward several competing fates: glycolysis, supporting ATP production, lactate formation and biosynthetic precursor generation; the cytosolic pentose phosphate pathway (PPP), producing NADPH and ribose 5-phosphate for redox control and nucleotide synthesis; glycogen synthesis and mobilization, providing a stress-buffering reservoir under hypoxia or nutrient fluctuation; or import into the endoplasmic reticulum (ER) through SLC37-family sugar-phosphate transporters, especially SLC37A4/G6PT and potentially SLC37A2 in selected tumour contexts. The model emphasizes that the oncological significance of the G6Pase system is not simply glucose production, but the routing of G6P between cytosolic and ER-localized metabolic compartments. In non-gluconeogenic tumours, increased or stabilized G6Pase system components may support stress adaptation, EMT, stemness, immune escape and therapy resistance. In gluconeogenic tissues such as liver and kidney, loss of G6PC may instead reflect or drive metabolic dedifferentiation, redirecting G6P away from glucose export toward biosynthetic, glycogenic and glycolytic programmes. Thus, G6P routing functions as a context-dependent metabolic rheostat rather than a pathway with a fixed oncogenic polarity. Throughout the schematic figures of this review (**Figures 1**–**4**), graphical conventions are used to distinguish levels of evidence. Solid arrows and filled symbols denote experimentally established relationships, whereas dashed arrows, open symbols and shaded boxes denote proposed, inferred or hypothetical interactions that await direct validation.

Early evidence from malignant gliomas supported this idea, as pharmacological and genetic interference with the G6P transporter G6PT/SLC37A4 modulated glioma-cell survival, migration and death, with chlorogenic acid and curcumin serving as functional probes (7, 8). These early studies also delineated an MT1-MMP/G6PT signalling axis that couples proMMP-2 activation to glioma-cell chemotaxis and to the decision between extracellular-matrix proteolysis and cell death, linked the transporter to sphingosine-1-phosphate-driven calcium signalling, and place its expression under hypoxia-inducible factor (HIF)-1α control (9–12). More recently, a coordinated G6PC3/SLC37A2/SLC37A4 signature has been associated with glioblastoma (GBM) progression, EMT, chemotaxis and a cancer-stem-cell phenotype (13). The proposal developed here is that these observations are not idiosyncratic to brain tumours but may reflect a more general principle: cancer cells exploit ER-localized G6P handling as a metabolic rheostat coordinating survival and aggressiveness (**Figure 1**, Key figure).

## 2 The glucose-6-phosphatase system: components and biological logic

The G6Pase system is best understood as a substrate-transport plus catalysis module spanning the ER membrane (5, 6). Three catalytic subunits hydrolyse G6P on the luminal face of the ER. G6PC1 (classically G6PC) is the gluconeogenic isoform expressed predominantly in liver, kidney and intestine (14, 15); G6PC2 is enriched in pancreatic islets and modulates glucose-stimulated insulin secretion (14); and G6PC3 is ubiquitously expressed and increasingly recognized for roles in neutrophil function (16–18) and, as discussed below, in tumour biology (14, 19). Because these catalytic subunits face the ER lumen, their activity depends on the delivery of cytosolic G6P across the ER membrane.

That delivery is mediated by the SLC37 family of sugar-phosphate antiporters. SLC37A4, the G6P transporter (G6PT), is the historically defined partner that couples cytosolic G6P import to luminal hydrolysis by G6PC/G6PC3 (5). Loss-of-function mutations in either SLC37A4 or G6PC3 cause defined congenital disorders, underscoring the obligate functional pairing of transport and catalysis for these two proteins (6). SLC37A1, SLC37A2 and SLC37A3 are less completely characterized because their canonical substrate specificities and coupling to the ER differ from those of SLC37A4. However, at least SLC37A2 is now emerging as a tumour-relevant actor in its own right (14, 19). This distinction matters for interpretation, since a tumour signature that includes SLC37A2 need not imply classical G6PT-type coupling; it may instead reflect a broader reprogramming of sugar-phosphate transport.

The biological logic that makes this system attractive in oncology is topological. By situating a hydrolytic step inside the ER lumen, the system creates a privileged compartment in which G6P can be consumed, sequestered or recycled independently of the cytosolic glycolytic pool (20). ER-resident handling of G6P intersects with hexose-6-phosphate dehydrogenase (H6PD)-linked luminal redox metabolism, calcium homeostasis, N-glycosylation, protein folding, and therefore the unfolded protein response (UPR) (21) and autophagy (22). A system that governs how much G6P enters this compartment, and what becomes of it, is thus positioned to influence far more than blood glucose.

**Figure 2:**
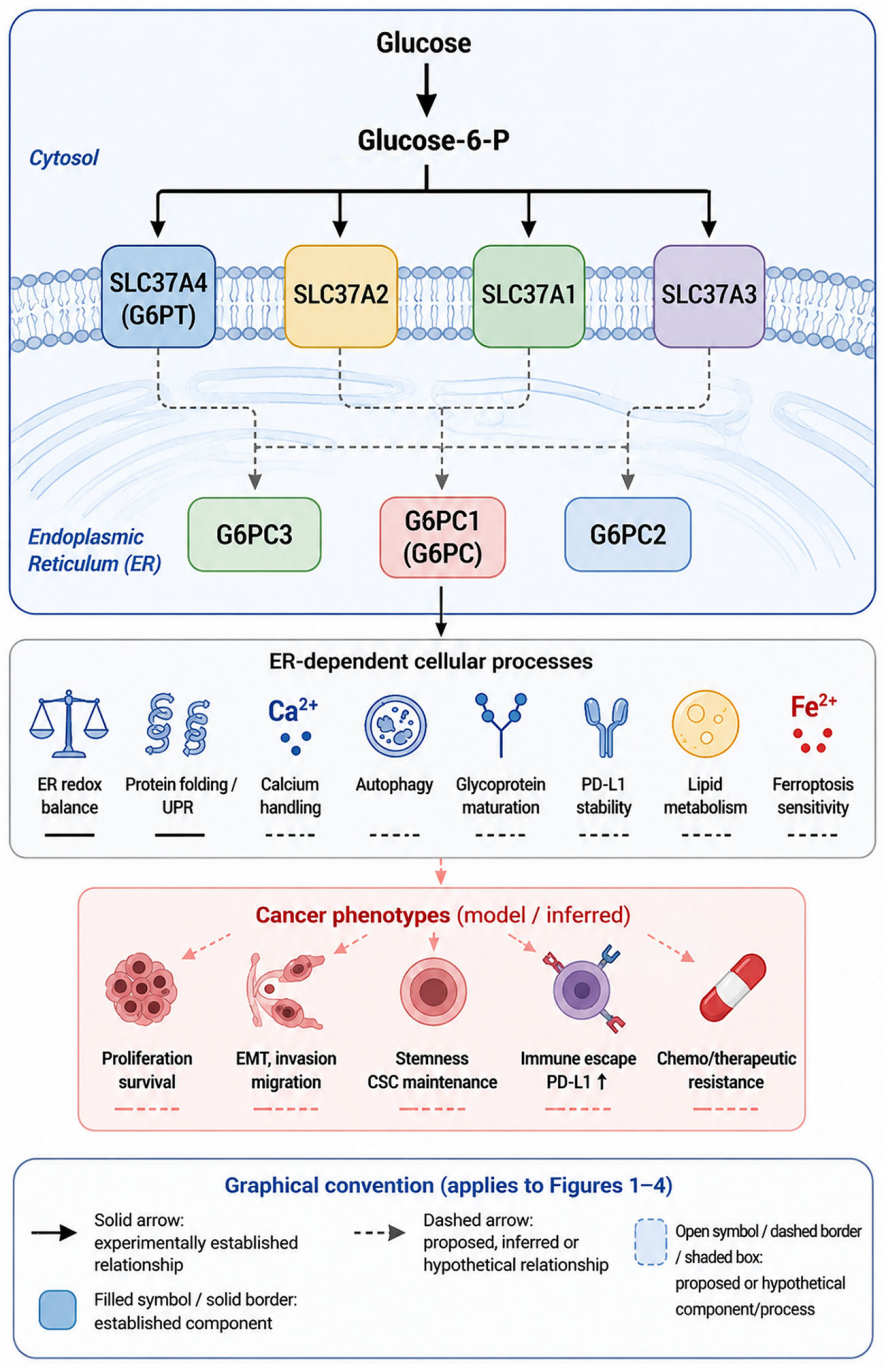
The glucose-6-phosphatase system as a metabolic rheostat of glucose/glucose-6-phosphate flux in cancer cells. Cancer cells import glucose and rapidly convert it into glucose-6-phosphate (G6P), a central metabolic intermediate positioned at the crossroads of glycolysis, the pentose phosphate pathway, glycogen metabolism, lipid biosynthesis and endoplasmic reticulum-associated metabolic control. The glucose-6-phosphatase system, composed of catalytic subunits G6PC1/G6PC, G6PC2 and G6PC3, together with SLC37A transporters including SLC37A1, SLC37A2, SLC37A3 and SLC37A4/G6PT, may regulate the compartmentalized routing of G6P toward the endoplasmic reticulum. In this context, ER-localized G6P handling may influence redox balance, calcium homeostasis, protein folding, autophagy, epithelial-mesenchymal transition, stemness, lipid metabolism, immune escape and therapeutic resistance. The figure proposes that cancer-associated dysregulation of G6Pase system components does not simply reflect altered glucose production, but rather a broader rewiring of intracellular G6P flux that may create context-dependent vulnerabilities for anticancer intervention.

A further mechanistic layer is provided by the ER-resident pentose phosphate machinery. In the ER lumen, H6PD can use imported G6P to generate luminal NADPH, thereby supporting compartment-specific redox reactions that are not directly interchangeable with the cytosolic NADPH pool (23). This is particularly relevant in cancer cells, where protein folding, disulfide-bond formation, calcium storage, lipid remodelling and ER-associated quality control are continuously challenged by hypoxia, nutrient limitation and oncogenic proteotoxic stress (20). From this perspective, G6P import into the ER should not be viewed only as a substrate-delivery step for glucose production, but as an entry point into a local redox and stress-adaptation circuit. Dysregulation of G6PC or SLC37A transporters may therefore alter the balance between productive ER adaptation and maladaptive UPR, with downstream consequences for autophagy, survival and therapy resistance (**Figure 2**).

## 3 Cancers in which the G6Pase system appears pro-tumoral

In tumours arising from tissues that are not natively gluconeogenic, up-regulation or stabilization of G6Pase system components is repeatedly linked to aggressive phenotypes. Five cancer types illustrate this pattern.

### 3.1 Glioblastoma

Glioblastoma (GBM) provided the founding observations. Early work showed that silencing or pharmacological targeting of G6PT/SLC37A4 altered glioma cell survival, migration and death, identifying the transporter as more than a passive conduit (7, 8). Mechanistically, gene silencing or pharmacological inhibition of either partner of this MT1-MMP/G6PT axis suppressed proMMP-2 activation and toggled glioma cells between an invasive programme and necrotic death, while blunting sphingosine-1-phosphate-induced calcium mobilization, ERK phosphorylation and chemotaxis. The transporter was further shown to be transcriptionally up-regulated by HIF-1α under the hypoxic conditions that typify GBM (9–12). This concept has since matured into a coordinated G6PC3/SLC37A2/SLC37A4 expression signature associated with GBM progression, hypoxic and TGF-β-driven programmes, EMT, chemotaxis and the maintenance of cancer-stem-cell properties (13). The GBM data are pivotal because they implicate transporter and catalytic components together, consistent with a flux-control rather than a single-enzyme model.

The glycogen dimension is particularly relevant for GBM and cancer-stem-like states. Glycogen is not simply an inert storage polymer; it can function as a stress-buffering reservoir that allows cancer cells to survive intermittent glucose deprivation, hypoxia and therapeutic pressure (4). In highly hypoxic tumours, a G6PC3/SLC37A4-centred ER flux may cooperate with glycogen turnover to maintain local G6P availability and sustain adaptive programmes such as autophagy, EMT and stemness. This interpretation is consistent with the observation that the G6PC3/SLC37A2/SLC37A4 signature is enriched in GBM progression and cancer-stem-cell phenotypes, and it suggests that glycogen metabolism should be experimentally integrated into future studies of G6Pase system dependency.

Independent lines of evidence reinforce the metabolic centrality of the G6Pase system in this tumour. G6Pase activity has itself been described as a key metabolic regulator of GBM invasion (24), and imaging-metabolic studies combining [^18^F]-fluoromisonidazole and [^18^F]-fluorodeoxyglucose positron-emission tomography have nominated G6PC3 as a candidate molecule in the hypoxic glucose metabolism of GBM (25). More recent computational work extends this picture, as machine-learning analysis of lactylation heterogeneity has identified G6PC3 as a prognostic target (26), while integrated single-cell and spatial-transcriptomic dissection of GBM has placed SLC37A3 among the genes contributing to a polygenic risk model of cellular heterogeneity (27). These reports are largely correlative, predictive or imaging-based rather than mechanistic, but their independent convergence on both catalytic (G6PC3) and transporter (SLC37A3) components is consistent with the coordinated, multi-component signature emphasised above and argues against a single-gene interpretation.

### 3.2 Breast cancer

In breast cancer, and particularly in the aggressive triple-negative subtype, G6PC is up-regulated in tumour samples and supports malignant glucose metabolism (28). A tRNA-derived fragment, tRFLys-CTT-010, is increased in triple-negative breast cancer (TNBC) and positively regulates G6PC; the resulting tRFLys-CTT-010/G6PC axis modulates lactate production and glycogen consumption, thereby promoting cancer-cell survival and proliferation. Silencing of G6PC reverses this oncogenic behaviour (28). These findings place G6PC-dependent handling of G6P within the broader thesis that ER-localized G6P metabolism may represent a tractable therapeutic target (20, 29), shifting the breast-cancer narrative from “more glucose” to “where G6P is routed”.

Recently, human SLC37A2 has also been proposed as a phospho-Ser294 progesterone-receptor (phospho-Ser294 PR) target gene (30). PR Ser294 phosphorylation is a common event in breast-cancer progression, and its activity is significantly associated with invasive lobular carcinoma. This observation is notable because it links a SLC37-family transporter, rather than a catalytic subunit, to hormone-receptor signalling, again pointing to broader sugar-phosphate-transport reprogramming as a feature of the breast-cancer phenotype.

### 3.3 Ovarian cancer

Ovarian cancer adds an immunometabolic dimension. Hexokinase domain-containing protein 1 (HKDC1) has been shown to promote tumour progression, lipid metabolism and immune escape by stabilizing G6PC/G6PC2, with downstream up-regulation of programmed death-ligand 1 (PD-L1) and functional suppression of T cells (31). This connects the G6Pase system not only to intrinsic proliferative signalling but also to the composition and competence of the tumour immune microenvironment, a link with obvious implications for combination with immunotherapy.

Earlier genetic and molecular analyses had already connected G6PC to glucose metabolism and to cell-cycle control in ovarian cancer, providing an initial link between this metabolic enzyme and proliferative regulation in the disease (32). The transporter arm of the system may also be relevant, since SLC37A3 has been highlighted among alternative-splicing events proposed as therapeutic targets in ovarian cancer (33). Together these observations suggest that, in the ovary as elsewhere, sugar-phosphate-transport reprogramming and not only catalytic-subunit stabilisation contributes to the malignant phenotype.

### 3.4 Gastric and colorectal cancer

In gastric cancer, a FOXO1/G6PC axis is associated with proliferation, metastasis and resistance to 5-fluorouracil, signalling through the PI3K/AKT/mTOR pathway (34). The recurrence of FOXO1 as an upstream regulator here and, with opposite directionality, in renal carcinoma (see below) is striking, and suggests that FOXO1 may act as a context-dependent node setting the sign of G6PC output.

Beyond this FOXO1/G6PC catalytic axis, gastrointestinal tumours also implicate the transporter side of the system. The SLC37 exchanger family has a well-characterised physiopathological role in sugar-phosphate handling (35), and SLC37A1 in particular has been reported as a candidate marker in these malignancies: it was identified among genome-wide markers proposed for the detection of circulating gastric-cancer cells (36) and is up-regulated in colorectal cancer, where its expression is associated with poor patient outcome and metastasis (37). Immune-focused analyses of gastric cancer have further embedded metabolic genes of this class within prognostic, immune-related signatures linked to clinical outcome and to cancer cachexia (38, 39). As elsewhere in this review, these reports are predominantly correlative and biomarker-oriented. Their value here is to widen the range of SLC37 components implicated across gastric and colorectal cancers, rather than to establish a single mechanistic route.

### 3.5 Cervical cancer

In cervical cancer, G6PC is again elevated and correlates with lymph-node metastasis, advanced clinical stage, recurrence and shorter survival. Functionally it stimulates proliferation, invasion, epithelial-mesenchymal transition (EMT) and angiogenesis through PI3K/AKT signalling (40). The convergence of EMT, angiogenesis and PI3K/AKT across cervical and gastric cancers points to a shared effector repertoire downstream of an over-active G6Pase system.

## 4 Cancers in which G6PC may be tumour-suppressive

It is important to resist the temptation to say that the G6Pase system would always be oncogenic. In tissues that are natively gluconeogenic, such as liver and kidney, the data point in the opposite direction, and this paradox is precisely what makes the system biologically interesting.

### 4.1 Hepatocellular carcinoma and hepatoma

In hepatocellular carcinoma, G6PC is reported to be decreased, negatively correlated with the proliferation marker Ki67 and lower in metastatic groups (41). Loss of this terminal gluconeogenic enzyme in hepatocytes plausibly reflects and may actively promote a loss of metabolic differentiation, with G6P retained for biosynthesis and glycogen accumulation rather than released as glucose.

A mechanistic window onto this tumour-suppressive direction is provided by the inherited loss of the gluconeogenic isoform in GSD type Ia (GSD-Ia), in which G6PC (G6Pase-α) deficiency strongly predisposes to hepatocellular adenoma and its malignant transformation to hepatocellular carcinoma. In a liver-specific G6pc-deficient mouse model that recapitulates the human disease, G6Pase-alpha deficiency produced sustained hepatic autophagy impairment, with progressive accumulation of the autophagy-specific substrate p62 and its phosphorylated form. This in turn drove coordinated activation of several tumour-promoting pathways, including Nrf2, mTORC1, beta-catenin and YAP, that together can initiate adenoma formation and favour conversion to carcinoma (42). The same study showed that carcinoma lesions contained significantly lower G6P and glycogen than the corresponding adenomas and overexpressed the M2 isoform of pyruvate kinase (PKM2), tying loss of the terminal gluconeogenic step to a further shift toward aerobic glycolysis and a more aggressive phenotype (42). Loss of G6PC in the hepatocyte is therefore not merely a passive marker of dedifferentiation. By impairing autophagic quality control and rerouting G6P away from glucose export, it can actively reconfigure the signalling and metabolic landscape toward malignancy, providing a concrete molecular route from deficiency of the gluconeogenic enzyme to liver cancer. These observations have direct counterparts in inherited G6Pase system deficiency. In a liver-specific GSD-Ia model, restoring G6Pase-alpha expression by rAAV-mediated G6PC gene transfer at the tumour-developing stage normalized glucose homeostasis and defective hepatic autophagy and prevented *de novo* adenoma/carcinoma initiation, even though it could not abrogate lesions that had already formed. In GSD-Ib, transporter (G6PT) deficiency was shown to impair hepatic autophagy through down-regulation of SIRT1/FoxO3a and LKB1/AMPK signalling, a further mechanism linking loss of G6Pase-system function to hepatocellular tumorigenesis (43, 44).

Hepatoma metabolism is nonetheless heterogeneous, and the residual capacity to hydrolyse G6P within the ER can vary markedly between models. In cultured hepatoma cells, G6Pase activity may remain high as HepG2 cells express G6Pase far more strongly than breast-cancer cells, and this is read out functionally as rapid dephosphorylation and efflux of 2-deoxy-2-[^18^F]fluoro-D-glucose ([^18^F]FDG), so that tracer accumulation saturates early and then declines rather than rising monotonically (45). In tumour-bearing mice, the same pattern was seen. HepG2 xenografts reached peak [^18^F]FDG signal within roughly 20–30 min and then lost signal, whereas G6Pase-low tumours and inflammatory lesions continued to accumulate tracer over time (45). This juxtaposition - clinical hepatocellular carcinoma and GSD-Ia models trending toward G6PC loss, yet well-differentiated hepatoma lines retaining brisk G6Pase-driven G6P export - is precisely what the routing model predicts. What determines the phenotype is where G6P is sent and whether it is hydrolysed within the ER, not simply whether the enzyme G6Pase is present. It also carries a concrete clinical corollary, since G6Pase-mediated [^18^F]FDG efflux can blunt the avidity of well-differentiated hepatoma on FDG-PET and confound the imaging-based distinction between tumour and inflammation, a caveat with direct relevance to staging (45).

Recent work on the gluconeogenic isoform G6PC1 reinforces and extends this tissue-contextual reading of hepatocellular carcinoma. Integrated multi-omics analysis of large patient cohorts has confirmed that G6PC1 is markedly down-regulated in HCC and that low expression independently predicts poorer survival, consistent with loss of the differentiated hepatocyte programme (46). The same work links G6PC1 to the lineage transcription factor HNF4A, to metabolic-reprogramming signatures, and to the composition of the tumour immune microenvironment and predicted immunotherapy response, thereby connecting loss of the terminal gluconeogenic step to both metabolic dedifferentiation and immune remodelling (46). Functional evidence points in the same direction since, in HCC models, a miR-494/G6pc axis drives glycolytic metabolic rewiring and modulates sensitivity to sorafenib, indicating that suppression of G6PC1 is not merely a passive marker of dedifferentiation but can actively reshape metabolism and therapy response (47). These studies have been integrated to better reflect the breadth of the current hepatocellular-carcinoma literature; we note, however, that several of these analyses are predominantly correlative or based on transcriptomic inference and therefore require direct mechanistic validation.

A complementary and, in the present context, particularly important perspective comes from tissue-specific analyses of the human phosphatome. A recent phosphatome-wide profiling of human protein phosphatases identified G6PC1 as one of the most liver-selective phosphatases in the genome and proposed it as a liver-cancer-selective biomarker, underscoring that the physiological and clinical significance of G6PC1 is inseparable from its restricted hepatic expression (48). Because G6PC1 is a highly tissue-specific hepatic metabolic phosphatase, its down-regulation in hepatocellular carcinoma is most coherently read not as a generic metabolic perturbation but as the erosion of a lineage-defining, organ-specific enzymatic identity. This tissue-specificity argument also helps to explain the apparent paradox running through this review, since the very catalytic activity that marks the differentiated hepatocyte behaves as a tumour-suppressive feature in the liver, whereas structurally related G6Pase system components are co-opted for pro-tumoral ER-flux control in non-gluconeogenic tissues.

Consistent with a broader remodelling of the sugar-phosphate-transport machinery in this disease, the transporter SLC37A3 has been proposed as a diagnostic and prognostic biomarker in hepatocellular carcinoma that is itself connected to glucose-metabolism regulation (49), and integrated multi-omics stratifications of the disease into proteomic subtypes have repeatedly recovered glucose- and gluconeogenesis-associated programmes among the discriminators of clinical behaviour (50). These observations sit within the wider framework of hepatocellular metabolic reprogramming, in which loss of terminal gluconeogenic and differentiated-hepatocyte functions accompanies a shift toward biosynthetic and glycolytic phenotypes (51). We stress, however, that several of these studies are explicitly biomarker-oriented or based on transcriptomic and proteomic inference; although they converge with the mechanistic data discussed above, the causal contribution of G6PC1 and SLC37A3 to hepatocarcinogenesis, as distinct from their value as markers of dedifferentiation, remains to be established by direct functional experiments.

### 4.2 Renal cell carcinoma

In renal cell carcinoma, G6PC was likewise described to be decreased, and inversely correlated with tumour stage, grade and immune infiltration, while its overexpression inhibited proliferation and migration. A FOXO1-G6PC axis was therefore proposed as a brake on progression (52). Here FOXO1-G6PC behaves as a tumour-suppressive module, the mirror image of the gastric FOXO1-G6PC circuit and of pro-tumoral G6PC-associated circuits in several non-gluconeogenic tumours. This reinforces the view that the direction of the system’s contribution is dictated by tissue context and by whether gluconeogenic differentiation is being maintained or abandoned. A mechanistic parallel to the hepatic situation is emerging in the kidney. In a GSD-Ia model, renal G6Pase-α deficiency drives persistently heightened autophagy with concomitant activation of both positive regulators (sirtuin-1, FoxO3a, AMPK) and the negative regulator mTOR, alongside elevated cyclin G1 and CDK5, a configuration that promotes maladaptive proximal-tubule dedifferentiation, G2/M arrest and profibrotic signalling in GSD-Ia nephropathy (53).

**Figure 3:**
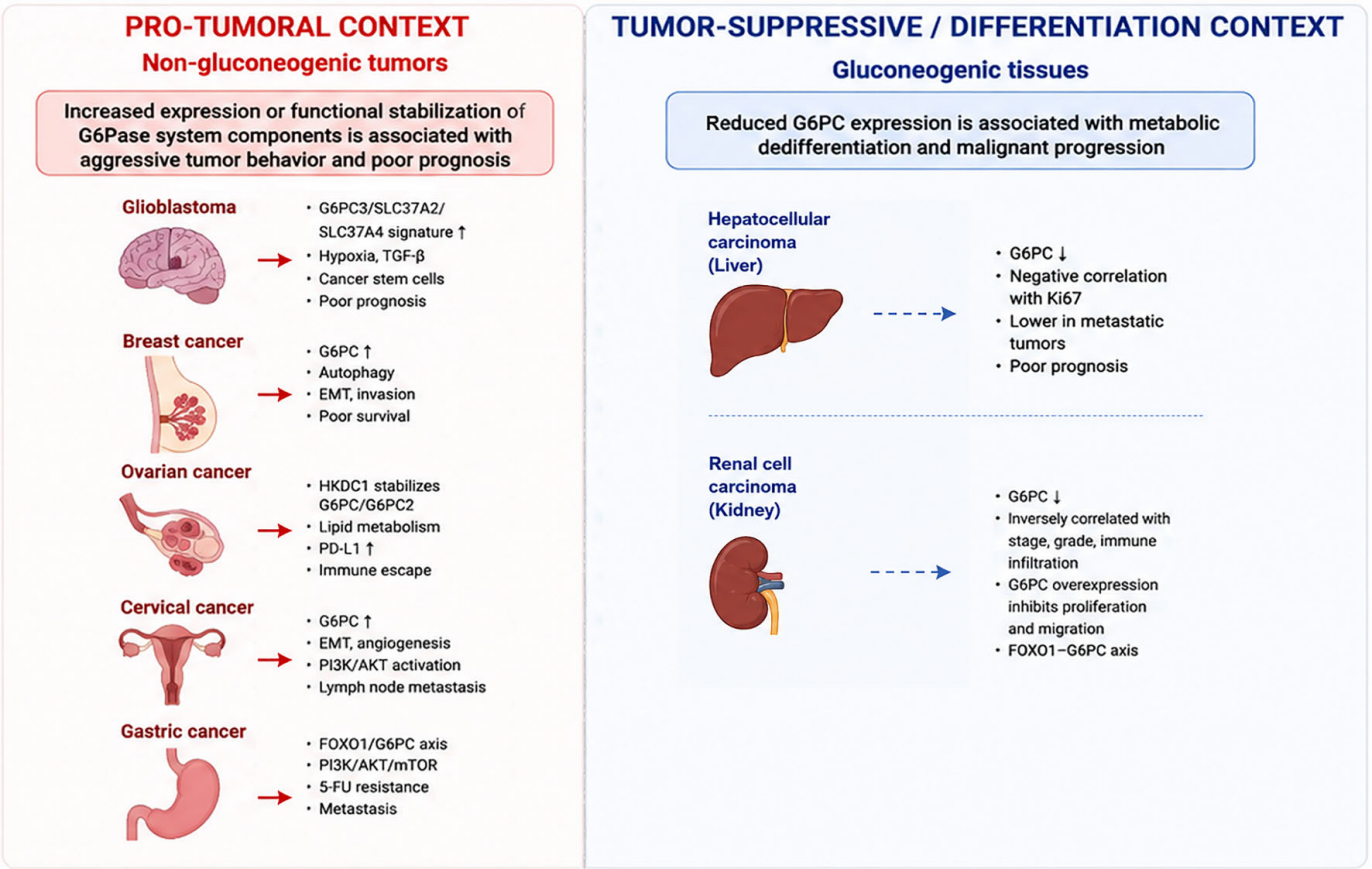
Context-dependent roles of the glucose-6-phosphatase system across cancer types. The glucose-6-phosphatase G6Pase system displays cancer-type- and tissue-context-dependent functions. In several non-gluconeogenic tumours, including glioblastoma, breast cancer, ovarian cancer, cervical cancer and gastric cancer, increased expression or functional stabilization of G6Pase system components is associated with aggressive phenotypes such as proliferation, epithelial–mesenchymal transition, invasion, autophagy, cancer stemness, immune escape and therapeutic resistance. In glioblastoma, the G6PC3/SLC37A2/SLC37A4 signature is linked to disease progression and acquisition of a cancer stem cell phenotype. In breast cancer, G6PC promotes malignant progression through autophagy-related mechanisms, while ER-localized G6P processing supports redox balance, protein folding and calcium homeostasis. In ovarian cancer, HKDC1-mediated stabilization of G6PC/G6PC2 promotes lipid metabolism and PD-L1-associated immune escape. In cervical and gastric cancers, G6PC contributes to EMT, angiogenesis, PI3K/AKT/mTOR activation and chemotherapy resistance. Conversely, in gluconeogenic tissues such as liver and kidney, reduced G6PC expression may contribute to metabolic dedifferentiation and malignant progression. In hepatocellular carcinoma and renal cell carcinoma, G6PC downregulation is associated with more aggressive disease features, and restoration or preservation of G6PC activity may restrain tumour progression. This apparent paradox suggests that the oncogenic or tumour-suppressive role of the G6Pase system depends on tissue lineage, metabolic differentiation state, intracellular G6P routing and the balance between cytosolic, glycogenic and ER-associated glucose metabolism. Rather than acting as a universal driver or suppressor, the G6Pase system should therefore be interpreted as a context-dependent regulator of cancer cell plasticity.

The unifying interpretation is not contradiction but compartmental logic. In gluconeogenic organs, high G6PC activity is a hallmark of the differentiated state where its loss accompanies dedifferentiation and malignancy. In non-gluconeogenic tissues, the same machinery is co-opted for ER-localized flux control that supports stress tolerance and aggressiveness. The system should therefore be read tissue-contextually, never as a single pathway with a fixed oncological polarity (**Figure 3**).

## 5 The glucose/glucose-6-phosphate flux as an actor in carcinogenesis

If the foregoing tumour-by-tumour evidence is to be coherent, the argument is not that cancer is about “more or less glucose”, it is that malignant cells exercise control over the fate of G6P, and that the G6Pase/SLC37A system is one of the principal devices through which that control can be exerted. It is important to frame this proposal appropriately. The ER-directed G6P-flux model advanced here is one of several non-exclusive interpretations of the available data, and it is intended to complement, rather than supplant, established frameworks centred on glycolytic flux, pentose-phosphate-driven redox control, glycogen dynamics and oncogenic signalling. More concretely, in several of the tumours discussed here these canonical frameworks, together with lineage-specific transcriptional programmes, may account for the reported associations at least as economically as ER-localized G6P handling; the ER-flux model should therefore be weighed against these alternatives rather than presumed to be the principal explanatory mechanism. Much of the supporting evidence is associative, derived from expression correlations, bioinformatics inference or perturbation studies in a limited number of models, and conflicting observations exist, most obviously the opposite directionality of G6PC expression across non-gluconeogenic and gluconeogenic tumours. In the sections that follow, we therefore try to distinguish experimentally established mechanisms from correlative associations and from frankly speculative models, and to flag explicitly where mechanistic uncertainty remains.

At any moment, a molecule of G6P may be committed to one of several mutually competitive routes including glycolysis for ATP and biosynthetic precursors, the cytosolic oxidative PPP for NADPH and nucleotide synthesis, glycogen synthesis for storage and stress buffering, ER import for luminal hydrolysis, or participation in an ER-resident pentose-phosphate limb that regulates luminal redox. The disposition of G6P among these routes feeds forward into redox poise, calcium handling, glycosylation and the UPR, autophagic flux, and lipid biosynthesis. Viewed this way, the G6Pase system functions less as a glucose-producing enzyme and more as a rheostat that sets the operating point of central carbon metabolism of the cell under duress.

This rheostat model accommodates the apparently divergent observations of the above sections. A tumour that up-regulates G6PC3/SLC37A4 may be tuning ER G6P handling to buffer proteotoxic and hypoxic stress, sustain autophagy, supply NADPH for redox defence, and favour EMT and stemness programmes. A hepatocyte that loses G6PC may be redirecting G6P away from glucose export and toward the biosynthetic and glycogenic pools that accompany dedifferentiation. In both cases the determining variable is G6P routing, not glucose abundance. The central, testable question for the field is therefore whether cancer cells use ER-localized G6P flux as a coordinating hub linking metabolism to organelle stress and phenotypic plasticity.

The same model also provides a plausible bridge between G6P routing and immune escape. A fraction of glucose-derived carbon can feed the hexosamine biosynthetic pathway, N-glycosylation and glycoprotein maturation, processes that are intimately dependent on ER integrity (54). In tumours in which G6Pase-system components enhance ER fitness, the consequence may extend beyond intrinsic survival to the stabilization and surface persistence of immune-regulatory proteins. The HKDC1-G6PC/G6PC2-PD-L1 axis described in ovarian cancer is consistent with this possibility and suggests that ER-localized glucose metabolism may indirectly shape immune-checkpoint biology (22). This does not imply that G6Pase components directly glycosylate PD-L1. Rather, they may sustain the metabolic and folding environment that permits immune-evasive proteins to be properly processed, stabilized and deployed at the tumour-cell surface.

ER-localized G6P flux may also intersect with mitochondrial stress responses (55). The ER is the major intracellular calcium reservoir and communicates with mitochondria through specialized contact sites (55). Perturbation of ER redox balance or calcium handling can therefore affect mitochondrial respiration, reactive oxygen species production, permeability-transition signalling and apoptosis sensitivity (56). In cancer cells, this ER-mitochondria axis may help explain why manipulation of G6PT/SLC37A4 or G6PC3 affects survival, migration and stress tolerance beyond what would be expected from a purely glycolytic mechanism. A related, still hypothetical, possibility is that G6Pase system inhibition could sensitize selected tumours to ferroptotic pressure by weakening NADPH-dependent antioxidant defences and increasing vulnerability to lipid peroxidation, especially in tumours that simultaneously rely on lipid biosynthesis and ER stress adaptation (57).

### 5.1 ER-localized G6P flux as a stress-adaptation hub: redox, calcium, glycosylation and ferroptosis

The emerging evidence invites a broader mechanistic model in which ER-localized G6P flux acts as a stress-adaptation hub rather than a simple glucose-releasing reaction. Once G6P is routed toward the ER, it may support luminal NADPH production through H6PD-dependent reactions (23), thereby influencing the redox environment required for protein folding, disulfide-bond formation and ER quality control (22). This local redox economy is likely to be particularly important in malignant cells, where oncogenic signalling, hypoxia and high secretory or membrane-protein demand impose a persistent proteotoxic burden.

This model also connects G6Pase system activity to calcium homeostasis and ER-mitochondria communication. Because ER calcium stores are sensitive to luminal redox conditions and protein-folding load, disruption of ER G6P handling may alter calcium release, mitochondrial reactive oxygen species production and apoptotic priming. These links may help explain why G6PT/SLC37A4 or G6PC3 manipulation affects survival, migration and stress tolerance in glioma models, and why ER-localized G6P metabolism emerges as a therapeutic vulnerability in breast cancer.

A third consequence concerns immune escape and glycoprotein processing. By preserving ER folding capacity and glucose-derived biosynthetic flux, the G6Pase system may support the maturation and membrane stability of immune-regulatory proteins. The ovarian cancer HKDC1-G6PC/G6PC2-PD-L1 axis is therefore notable because it links sugar-phosphate metabolism, lipid rewiring and immune-checkpoint expression in a single oncogenic circuit (31). Finally, because ER redox and lipid metabolism are tightly connected (58), G6Pase system inhibition could, in selected tumours, increase vulnerability to ferroptosis by limiting NADPH-dependent antioxidant buffering and enhancing lipid-peroxidation stress. These proposed connections should be tested directly using compartmentalized G6P and NADPH sensors, ER calcium reporters, glycoproteomic analyses and ferroptosis-rescue experiments.

### 5.2 Comparative metabolic consequences and integration with canonical glycolytic and pentose-phosphate regulators

A direct comparison of the metabolic consequences of G6Pase system activity in its pro-tumorigenic versus tumour-suppressive settings helps to make the routing model concrete. In non-gluconeogenic tumours that up-regulate or stabilize G6PC3/SLC37A4, or, in ovarian cancer, G6PC/G6PC2, increased import and luminal handling of G6P is proposed to withdraw a fraction of the cytosolic pool from immediate glycolytic commitment, to in part feed H6PD-dependent luminal NADPH production, and to support glycogen turnover as a stress buffer. The predicted net effect would then be enhanced redox and proteostatic resilience, sustained autophagy, and favouring of EMT, stemness and immune-evasive programmes under hypoxic and proteotoxic stress. In gluconeogenic tissues such as liver and kidney, the same machinery operates in the opposite regime, where loss of G6PC removes the terminal dephosphorylation step, so that G6P is retained in the cytosol and redirected toward glycolysis, with up-regulation of PKM2, and toward the PPP, glycogen and biosynthetic pools, resulting in impaired autophagic quality control and in metabolic dedifferentiation. The contrast is therefore not one of opposite pathways but of opposite flux set points, where in both settings the determining variable is where G6P is routed and whether it is hydrolysed within the ER.

Framed as a branch-point competition, ER import of G6P is in direct kinetic competition with the cytosolic fates of the metabolite. Carbon committed to ER handling or to glycogen is transiently unavailable for glycolytic ATP and lactate production, for oxidative-PPP generation of NADPH and ribose 5-phosphate, for the lipogenic acetyl-CoA pool, and for the hexosamine biosynthetic pathway (HBP) that supplies UDP-GlcNAc for N- and O-glycosylation. Because the HBP and ER-localized glycosylation are themselves dependent on ER fitness, routing of G6P toward the ER may indirectly reinforce glycoprotein maturation even as it competes for upstream carbon, providing a plausible metabolic link between G6P partitioning and the surface stability of immune-regulatory glycoproteins. These competing fluxes are interdependent rather than strictly additive, and their relative weighting is expected to vary with tissue lineage, oncogenic background and microenvironmental stress.

This branch-point logic can be positioned explicitly relative to the canonical glycolytic and PPP regulators that dominate cancer metabolism models. Hexokinase-2 (HK2) sets the entry rate of glucose into the G6P pool and thus the total flux available for partitioning; phosphofructokinase (PFK) and the M2 isoform of pyruvate kinase (PKM2) gate downstream glycolytic commitment, with PKM2 in its low-activity state favouring accumulation of upstream intermediates that can be diverted toward the PPP, glycogen or ER handling; and glucose-6-phosphate dehydrogenase (G6PD) commits G6P to the cytosolic oxidative PPP for NADPH production (59). The G6Pase/SLC37A system is best viewed as an additional, ER-localized drain on the same shared G6P pool that operates in parallel with these enzymes rather than in isolation, and resulting in a high-HK2, low-PKM2-activity, high-G6PD pro-tumoral configuration that accumulates G6P and that is expected to maximize the substrate available for SLC37A4-dependent ER import, whereas unrestricted glycolytic commitment would limit it. The recurrent appearance of PKM2 in the GSD-Ia hepatocarcinogenesis paradigm, of HKDC1, a hexokinase-domain protein, as a stabilizer of G6PC/G6PC2 in ovarian cancer, and of PPP-linked redox control across these tumours is consistent with such coupling. We emphasize, however, that the quantitative interaction between the G6Pase system and HK2, PFK, PKM2 and G6PD has yet to be assessed directly, and that establishing it will require compartmentalized flux analysis rather than expression correlation alone.

Finally, the polarity of these consequences is inseparable from the highly tissue-specific physiology of the individual components. G6PC1 is the gluconeogenic isoform of liver, kidney and intestine, and its loss in hepatocellular and renal carcinoma is most parsimoniously read as dedifferentiation away from a glucose-exporting identity. G6PC2 is largely restricted to pancreatic islets, where it tunes glucose-stimulated insulin secretion, so that its appearance in the ovarian HKDC1 axis represents a context distinct from its native physiology. G6PC3 is ubiquitously expressed, with established roles in neutrophil function and an emerging, context-dependent role in epithelial tumours. On the transport side, SLC37A4/G6PT is the obligate, genetically validated partner of G6PC/G6PC3, whereas SLC37A1 to SLC37A3 differ in substrate specificity and ER coupling, so that a tumour signature including SLC37A2 need not imply classical G6PT-type catalysis and may instead reflect broader sugar-phosphate-transport reprogramming. These biological differences mean that identical changes in transcript level can carry opposite functional meaning depending on organ of origin, and they caution against treating the G6Pase system as a single, uniformly polarized entity.

## 6 The G6Pase system in tumour-associated immune cells: macrophages and neutrophils

The discussion so far has treated the G6Pase system as a property of the malignant cell itself. Yet solid tumours are not purely epithelial masses. Their progression is shaped by infiltration where myeloid cells predominate, and tumour-associated macrophages and neutrophils are among the most abundant non-malignant constituents of that infiltrate. It is therefore relevant, although still indirect for oncology, that the same G6Pase/G6PT machinery is known to govern the differentiation and metabolic programming of these cell types. The clearest evidence comes from GSD type Ib (GSD-Ib), in which the functional loss of the G6P transporter SLC37A4/G6PT, the partner that couples cytosolic G6P import to luminal hydrolysis by by G6Pase-β/G6PC3, produces not only classical neutropenia but a broader failure of myeloid maturation. In a human monocytic model and in G6pt-deficient mice, G6PT deficiency impaired the differentiation of monocytes into macrophages as the cells over-proliferated, adhered and spread poorly, expressed less of the maturation marker CD11b, and were locked in an aberrant metabolic state characterized by enhanced glycolysis with a reduced glycolytic reserve and impaired mitochondrial oxidative phosphorylation, the latter traced to elevated PDK1 and pyruvate-dehydrogenase phosphorylation (60). Functionally, these G6PT-deficient monocytes also displayed a blunted inflammatory-cytokine response despite their elevated glucose consumption, indicating that the transporter shapes not only whether a macrophage forms but how it behaves once formed (60).

A parallel logic operates in the neutrophil lineage. G6PT deficiency arrests neutrophil maturation in the bone marrow, and in a promyelocyte model, the block was attributed to two converging defects: abnormal lipid metabolism that delayed metabolic reprogramming required for differentiation, and reduced nuclear transcriptional activity of PPARγ, a receptor essential for the cell-cycle exit and gene-expression programme of granulocytic maturation (61). Here too the cells drifted toward excessive, poorly coupled mitochondrial respiration with depleted lipid droplets and up-regulated PPARα, while repressors of PPARγ such as PU.1 and SIRT1 accumulated and ERK1/2 dephosphorylation was delayed (61). The recurring theme across both lineages is that an intact ER G6P-handling system is required for the orderly metabolic switch between glycolysis, fatty-acid oxidation and oxidative phosphorylation that accompanies myeloid differentiation, and that its loss freezes the cell in an immature, metabolically inflexible state.

**Figure 4:**
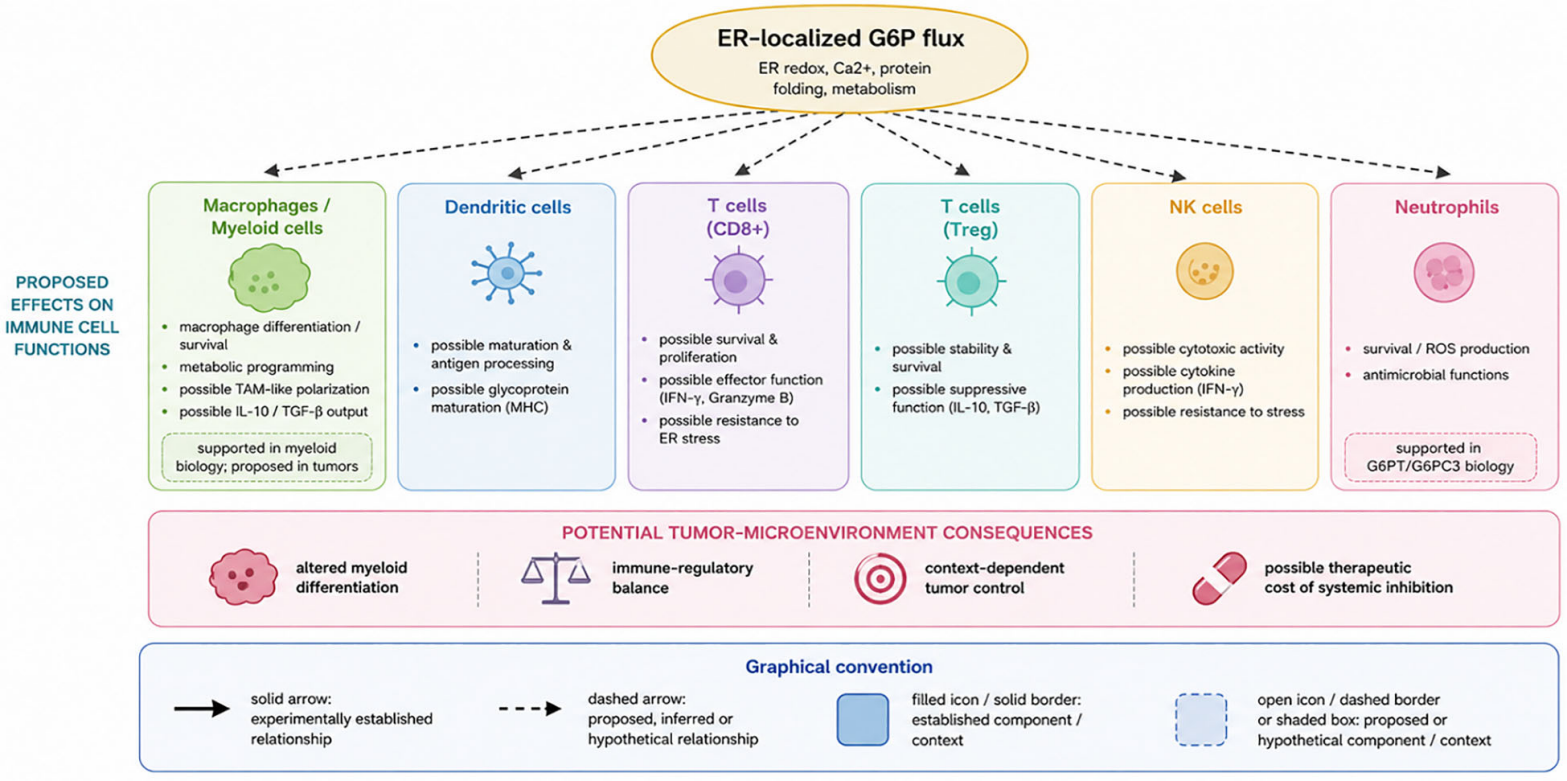
ER-localized glucose-6-phosphate flux may modulate immune cell functions in the tumour microenvironment. ER-localized glucose-6-phosphate (G6P) flux acts as a metabolic and functional hub that influences multiple dimensions of anti-tumour immunity. By shaping ER redox homeostasis, calcium signalling, protein folding, glycosylation, and cellular metabolism, this system can support the survival, differentiation, and effector activity of distinct immune cell populations. In macrophages and myeloid cells, ER-localized G6P flux may promote ER fitness and survival, favour M2/TAM polarization, and enhance the production of immunosuppressive cytokines such as IL-10 and TGF-β. In dendritic cells, it may contribute to maturation, antigen processing, MHC-dependent presentation, and glycoprotein maturation. In CD8^+^ T cells, it supports survival, proliferation, effector functions, including IFN-γ and granzyme B production, and resistance to ER stress. In regulatory T cells, it promotes stability, survival, and suppressive activity. It may also enhance NK-cell cytotoxicity, cytokine production, and stress resistance, while sustaining neutrophil survival, ROS production, and antimicrobial functions. Overall, ER-localized G6P flux may strengthen selected immune functions, maintain immune-regulatory balance, contribute to tumour growth control, and shape the quality of anti-tumour immunity within the tumour microenvironment.

For oncology framed around G6P routing, this myeloid dimension carries two implications. First, it widens the territory in which the G6Pase system may influence tumour behaviour if transporter- and catalysis-dependent G6P handling sets the differentiation state and effector competence of macrophages and neutrophils, then the system is positioned to modulate the tumour immune microenvironment from within the infiltrate itself, and not only from within the cancer cell. This connects to the immune-infiltration and immune-escape observations noted earlier through the inverse correlation between G6PC and immune infiltration in renal carcinoma (46), and the HKDC1-G6PC/G6PC2-PD-L1 axis in ovarian cancer (31), and suggests that the net immunological effect of G6Pase system activity will depend on how it is distributed between the malignant and immune compartments. Second, it sharpens the note of caution already implicit for therapy. Because loss of G6PT or of G6Pase-β/G6PC3 is precisely what produces neutropenia and defective macrophage and neutrophil function in GSD-Ib (60, 61), systemic inhibition of the transporter or of the G6Pase-β catalytic subunit risks reproducing that immunodeficiency, potentially crippling the very myeloid defences that help restrain tumour growth and infection. Any strategy aimed at G6P routing must therefore weigh a possible anti-tumour benefit within the cancer cell against an immunosuppressive cost within the infiltrate, reinforcing the case for tumour-selective, context-restricted intervention developed in the next section (**Figure 4**).

## 7 Pharmacological inhibitors: promise and prudence

If G6P routing is a vulnerability, can it be drugged? Proof-of-concept agents are already familiar from glioma work, where chlorogenic acid and curcumin were used to interrogate G6PT/SLC37A4-dependent phenotypes (7, 8, 29). Beyond these dietary polyphenols, semisynthetic analogs of the polyketide mumbaistatin have been characterized as more potent G6PT inhibitors that curtail HIF-1α–dependent survival under hypoxia and display antiangiogenic activity in brain endothelial cells, offering an additional chemical scaffold for targeting the transporter (12, 62). These compounds are valuable as functional probes and as starting points for medicinal chemistry, but they are pleiotropic and not isoform-selective, and they should not be over-interpreted as clinical candidates in their current form.

A credible therapeutic programme would have to satisfy several conditions. First, isoform selectivity is essential: G6PC1, G6PC2 and G6PC3 have distinct tissue distributions and physiological roles, and indiscriminate inhibition risks metabolic toxicity, particularly hypoglycaemia and neutrophil dysfunction. Second, tumour and tissue specificity are critical because the system appears tumour-suppressive in liver and kidney. A systemically active inhibitor could therefore be harmful precisely where the pathway is protective. Third, rational combination is likely to matter. Given connections of the G6Pase system to ER stress, autophagy, glycolysis, PI3K/AKT/mTOR signalling and immune evasion, the most promising strategies may pair G6Pase system inhibition with UPR or autophagy modulators, PI3K/AKT/mTOR inhibitors or, in light of the HKDC1/PD-L1 axis, immune-checkpoint blockade.

A note of caution should temper the reference to therapeutic vulnerability in the title and throughout this Review. Strategies discussed above have to be supported by direct clinical evidence. The available data rest on functional probes, pleiotropic natural compounds, indirect metabolic interventions and preclinical models, and isoform- and tumour-selective inhibitors of the G6Pase system suitable for clinical testing do not currently exist. We therefore distinguish between a small number of experimentally supported opportunities, chiefly transporter-level interference in tumour types where the system is most clearly pro-tumoral, and the broader set of combination strategies, which at present represent future research directions rather than validated therapeutic options.

Targeting the transporter rather than the catalytic subunit also deserves particular attention. Because SLC37A4/G6PT gates substrate entry into the ER, its inhibition may reduce or constrain ER luminal G6P flux without requiring isoform catalytic selectivity, and the genetic precedent of GSD-Ib offers a partial guide to the expected on-target physiology (60, 61). The emerging tumour relevance of SLC37A2 raises the further possibility of transporter-level vulnerabilities that are tumour-enriched and therefore better tolerated than inhibition of the housekeeping catalytic machinery.

## 8 Concluding remarks

### 8.1 Toward an oncology of glucose-6-phosphate flux

The paradoxical behaviour of G6PC across tumour types may be best interpreted as a polarity switch rather than a contradiction. In liver and kidney, G6PC expression is part of the differentiated metabolic identity of the tissue. Its loss may therefore mark or promote metabolic dedifferentiation, intracellular retention of G6P, glycogen imbalance and biosynthetic redirection. In non-gluconeogenic tumours, however, expression or stabilization of G6Pase system components may create a neo-functional ER flux module that supports stress tolerance and malignant plasticity. FOXO1 may sit at the centre of this switch. Depending on lineage, chromatin state, nutrient context and signalling background, FOXO1-G6PC circuits may either restrain progression by maintaining differentiation or promote malignancy by coupling G6P flux to autophagy, PI3K/AKT/mTOR signalling and therapy resistance.

The accumulating evidence suggest that the role of the G6Pase system needs to be reconsidered. The G6Pase system should no longer be filed under “inherited genetic disease of the liver, kidney and neutrophils”. In cancer, it behaves as an interface between metabolism, organelle stress and phenotypic plasticity: a compartmentalized controller of G6P flux at the ER. Its components are differentially deployed across tumour types, being pro-tumoral when up-regulated in glioblastoma and in breast, ovarian, gastric and cervical cancers, and tumour-suppressive when lost in the gluconeogenic settings of liver and kidney (**Table 1**).

**Table 1:** The glucose-6-phosphatase system across cancer types.

Cancer	Components implicated	Trend	Associated phenotype
Glioblastoma	G6PC3, SLC37A2, SLC37A4	↑	Progression, EMT, chemotaxis, stemness, cancer stem cells
Breast/TNBC	G6PC; ER G6P flux	↑	TNBC progression; lactate production; glycogen consumption; survival/proliferation; ER stress
Ovarian	G6PC/G6PC2 stabilized by HKDC1	↑	Lipogenesis, PD-L1, immune escape, invasion
Gastric	FOXO1/G6PC	↑	Metastasis, 5-FU resistance, PI3K/AKT/mTOR
Cervical	G6PC	↑	EMT, angiogenesis, invasion, recurrence
Liver/HCC	G6PC	↓	Loss of metabolic differentiation; autophagy impairment; p62/Nrf2/mTORC1/beta-catenin/YAP; PKM2-driven glycolysis (GSD-Ia paradigm); possible metastatic/poor-prognosis association
Kidney/RCC	FOXO1-G6PC	↓	G6PC proposed as a brake on progression

Summary of G6Pase-system components implicated in selected cancers, their reported expression or stabilization trends, and associated phenotypes. Increased or stabilized components generally support aggressive behaviour in non-gluconeogenic tumours, whereas decreased G6PC expression in liver and kidney cancers may reflect loss of metabolic differentiation and a tumour-suppressive function. The table highlights the context-dependent polarity of G6Pase system activity in cancer. Rows marked ↑ (increased/stabilized) pro-tumoral indicate contexts in which the system appears to support tumour progression. Rows marked ↓ (decreased/lost) tumour-suppressive indicate gluconeogenic tissues in which G6PC appears protective or differentiation preserving.

Three priorities for future work on the G6Pase system in cancer follow. Mechanistically, the field needs direct measurement of ER-localized G6P handling and of how G6PC3, SLC37A4/G6PT and the SLC37A2 axis are wired into redox, calcium, the UPR and autophagy. Clinically, expression of the catalytic subunits and transporters should be evaluated as tissue-contextual prognostic and predictive biomarkers rather than as a single marker. Therapeutically, the goal is isoform- and tissue-selective, combination-ready interruption of G6P routing, most plausibly at the transporter level, tested first where the pathway is best supported as pro-tumoral.

In summary, the most compelling interpretation of the emerging literature is that the G6Pase system acts as a context-dependent organizer of intracellular G6P fate. Its importance in cancer may result from the ability to connect central carbon metabolism with ER redox balance, calcium homeostasis, protein folding, autophagy, glycoprotein maturation, immune escape and lipid-peroxidation. This view generates testable predictions. Tumours with high G6PC3/SLC37A4 or HKDC1-G6PC/G6PC2 activity should display measurable dependency on ER-localized G6P handling, whereas liver and kidney tumours with G6PC loss should exhibit features of metabolic dedifferentiation. The next step is therefore not only to catalogue expression of G6Pase system genes, but also to map their compartmental fluxes and stress-adaptation outputs in living cancer cells.

## Glossary

5-FU: 5-fluorouracil
AKT: protein kinase B
AMPK: AMP-activated protein kinase
CSC: cancer stem cell
EMT: epithelial–mesenchymal transition
ER: endoplasmic reticulum
FDG: 2-deoxy-2-[^18^F]fluoro-D-glucose
FDG-PET: 2-deoxy-2-[^18^F]fluoro-D-glucose positron emission tomography
FOXO1: forkhead box O1
G6P: glucose-6-phosphate
G6Pase: glucose-6-phosphatase
G6PC: glucose-6-phosphatase catalytic subunit
G6PC1: glucose-6-phosphatase catalytic subunit 1
G6PC2: glucose-6-phosphatase catalytic subunit 2
G6PC3: glucose-6-phosphatase catalytic subunit 3
G6PD: glucose-6-phosphate dehydrogenase
G6PT: glucose-6-phosphate transporter
GBM: glioblastoma
GSD: glycogen storage disease
GSD-Ia: glycogen storage disease type Ia
GSD-Ib: glycogen storage disease type Ib
H6PD: hexose-6-phosphate dehydrogenase
HBP: hexosamine biosynthetic pathway
HCC: hepatocellular carcinoma
HIF1-α: hypoxia-inducible factor 1 alpha
HKDC1: hexokinase domain-containing protein 1
LKB1: liver kinase B1
mTOR: mechanistic target of rapamycin
mTORC1: mechanistic target of rapamycin complex 1
NADPH: nicotinamide adenine dinucleotide phosphate, reduced form
PD-L1: programmed death-ligand 1
PDK1: pyruvate dehydrogenase kinase 1
PI3K: phosphoinositide 3-kinase
PKM2: pyruvate kinase M2
PPP: pentose phosphate pathway
PPARα: peroxisome proliferator-activated receptor alpha
PPARγ: peroxisome proliferator-activated receptor gamma
PR: progesterone receptor
RCC: renal cell carcinoma
SIRT1: sirtuin 1
SLC37A1: solute carrier family 37 member 1
SLC37A2: solute carrier family 37 member 2
SLC37A3: solute carrier family 37 member 3
SLC37A4: solute carrier family 37 member 4
TGF-β: transforming growth factor beta
TNBC: triple-negative breast cancer
UPR: unfolded protein response
YAP: yes-associated protein
